# Clinical Applications of S53P4 Bioactive Glass in Bone Healing and Osteomyelitic Treatment: A Literature Review

**DOI:** 10.1155/2015/684826

**Published:** 2015-10-04

**Authors:** N. A. P. van Gestel, J. Geurts, D. J. W. Hulsen, B. van Rietbergen, S. Hofmann, J. J. Arts

**Affiliations:** ^1^Orthopaedic Biomechanics Group, Department of Biomedical Engineering, Eindhoven University of Technology, P.O. Box 513, 5600 MB Eindhoven, Netherlands; ^2^Department of Orthopaedic Surgery, CAPHRI Research School, Maastricht University Medical Centre, P.O. Box 5800, 6229 HX Maastricht, Netherlands; ^3^Institute for Complex Molecular Systems, Department of Biomedical Engineering, Eindhoven University of Technology, P.O. Box 513, 5600 MB Eindhoven, Netherlands; ^4^Institute for Biomechanics, Swiss Federal Institute of Technology Zürich (ETHZ), Vladimir-Prelog-Weg 1-5/10, 8093 Zürich, Switzerland

## Abstract

Nowadays, S53P4 bioactive glass is indicated as a bone graft substitute in various clinical applications. This review provides an overview of the current published clinical results on indications such as craniofacial procedures, grafting of benign bone tumour defects, instrumental spondylodesis, and the treatment of osteomyelitis. Given the reported results that are based on examinations, such as clinical examinations by the surgeons, radiographs, CT, and MRI images, S53P4 bioactive glass may be beneficial in the various reported applications. Especially in craniofacial reconstructions like mastoid obliteration and orbital floor reconstructions, in grafting bone tumour defects, and in the treatment of osteomyelitis very promising results are obtained. Randomized clinical trials need to be performed in order to determine whether bioactive glass would be able to replace the current golden standard of autologous bone usage or with the use of antibiotic containing PMMA beads (in the case of osteomyelitis).

## 1. Introduction

Bone graft substitutes are commonly used to replace and regenerate bone lost due to trauma, infection, disease, or for stability around implanted devices [1]. Current generation biomaterials are designed to stimulate specific cellular responses at the molecular level. This generation of biomaterials is both bioactive and degradable and might be osteoconductive or osteoinductive [2]. Bioactivity refers to any interaction or effect that materials have on cells to activate specific responses [3, 4]. Such a promising biomaterial is bioactive glass, an osteostimulative material that is currently used as bone graft substitute and in the treatment of osteomyelitis. Osteostimulation refers to osteoblast cell recruitment and/or differentiation and osteoblast activation to produce new bone in a bony environment [5]. Osteostimulation should not be confused with osteoinduction, which is the ability of materials to recruit stem cells to differentiate into bone forming cells and form ectopic bone. Also it should not be confused with osteoconduction, which is the possibility for bone to grow along the material, in other words only providing a scaffold for bone formation [6]. Bioactive glass is an osteostimulative material; thus it is osteoconductive and serves as a scaffold for bone formation* in vivo*, but it is not fully osteoinductive since it will only form new orthotopic bone (whereas osteoinductive materials are able to form ectopic bone).

**Figure 1:**
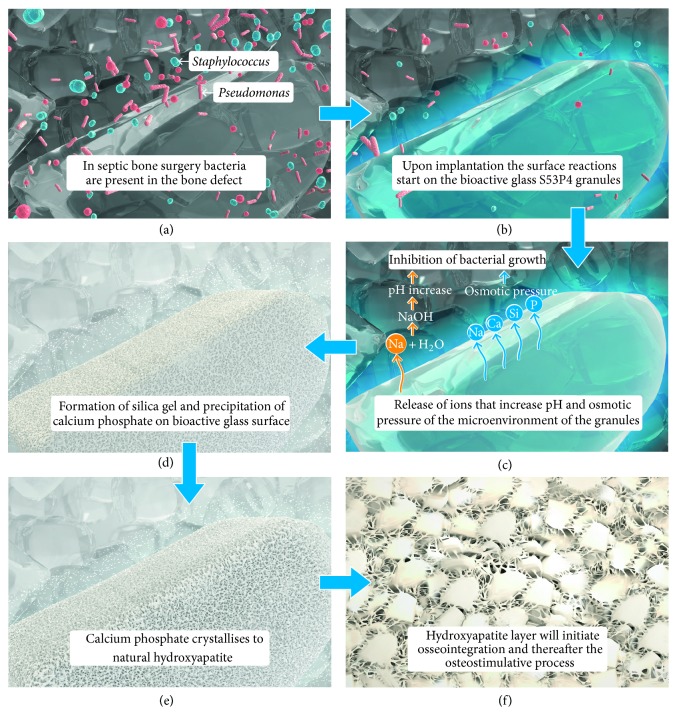
An illustration of the surface reactions of bioactive glass after implantation. When bioactive glass is implanted in a septic bone defect it will exchange alkali from the glass surface with the hydronium in the surrounding microenvironment, which will increase the local pH. The release of ions of the glass surface will also increase the osmotic pressure locally. A silica gel layer will be formed near the glass surface to which amorphous calcium phosphate precipitates and subsequently will crystallise into natural hydroxyapatite. The hydroxyapatite will induce the osteostimulative effect by activating osteogenic cells. This figure was kindly provided by BonAlive Biomaterials Ltd.

After implantation of S53P4 bioactive glass, surface reactions ensure deposition of a calcium phosphate layer when exposed to (body) fluid. Sodium, silica, calcium, and phosphate ions are released from the surface and increase the local pH and osmotic pressure. Thereafter, a silica gel layer is formed on the glass surface, and amorphous calcium phosphates precipitate on this layer. These amorphous structures then crystalize to natural hydroxyapatite, which starts the activation of osteoblasts for the formation of new bone [7–9]. This mechanism of action is illustrated in Figure 1. Because of the continuous reactions and layer formation, the glass will finally be absorbed [7, 8]. The surface reactions not only are beneficial for the formation of new bone but also ensure that bioactive glass contains antibacterial properties and potentially promotes angiogenesis [10–17].

Various compositions of bioactive glasses have been developed because of their composition-dependent promising bone forming properties, antibacterial properties, and degradability. This review focuses on S53P4 bioactive glass (BonAlive Biomaterials Ltd., Turku, Finland), with the specific composition (by weight) of 53% SiO_2_, 4% P_2_O_5_, 23% Na_2_O, and 20% CaO [8, 9]. This composition is used increasingly in clinical practice in various bone graft applications and in treatment of osteomyelitis. S53P4 bioactive glass is indicated to facilitate and stimulate bone formation and bone defect healing and to have an antibacterial effect in various applications [9]. S53P4 bioactive glass is mostly used in a granular form (0.8–3.15 mm), but sometimes it is used in the form of nonporous plates or discs in various shapes. The aim of this review is to provide a literature overview of reported clinical outcomes in different applications of S53P4 bioactive glass. The focus is on its application in bone defect healing and osteomyelitis treatment.

## 2. Clinical Application of S53P4 Bioactive Glass in Craniofacial Surgery

The first reported applications of S53P4 bioactive glass in clinical practice were performed in craniofacial surgery (Table 1). Conventional reconstructions of orbital floors and other facial bones are usually performed with autologous bone or cartilage grafts [39, 40]. However, interest has arisen in the use of alloplastic materials to eliminate the disadvantages of autologous graft usage such as donor site morbidity and limitations in size, shape, quality, or quantity [39].

Suominen and Kinnunen [18] reported defect reconstruction of facial bones with bioactive glass granules and plates. S53P4 bioactive glass granules and plates were implanted in 36 sites in thirteen patients. Granules were used in subperiosteal pockets of facial bones and to obliterate frontal sinuses, whereas plates were used for the reconstruction of orbital walls. Prospective middle face radiographic and computed tomographic (CT) imaging results were promising, showing that the material was well tolerated by the body. Moreover, no refractures were observed up to one year postoperatively. Subsequently, further trials were published, demonstrating additional positive bone healing results with S53P4 bioactive glass in facial reconstructions. For the use of bioactive glass plates, used in orbital floor reconstructions, good clinical outcomes have been reported. No complications due to the use of bioactive glass have been observed in standard postoperative, clinical examinations, CT and/or magnetic resonance (MR) images, and radiographs. Surprisingly, the bioactive glass plates did not show degradation [19–21]. It is not completely clear why degradation has not been observed. It has been hypothesized that no degradation could have taken place because of the structure of the plates, since they are solid and rigid, and pores are lacking. In plates, the surface area per volume is much smaller than in granules, such that the reactivity in plates is less than in granules. Peltola et al. [20] described only slight new bone formation after two years in the lower surface of the bioactive glass plates by histological evaluation of two cases. As already mentioned, the lack of bone formation is probably caused by the fact that plates are not porous and therefore obstruct the osteostimulative effect of the bioactive glass. In addition, this may hamper mechanical interlock with surrounding bone tissue [4, 41]. More recently, Stoor et al. [21] studied the performance of a new drop shaped S53P4 bioactive glass plate to repair orbital floor fractures in a prospective clinical study, with two years of follow-up in twenty patients. No adverse tissue reaction was associated with the material, and due to the anatomical drop shape, the implants could successfully maintain the orbital volume while compensating for the retrobulbar adipose tissue atrophy. Again, no degradation was observed in this study.

In frontal sinus obliteration, small size bioactive glass granules (0.5–0.8 and 0.8–1 mm) have been used for treatment in 24 patients suffering from chronic frontal sinusitis [22]. During follow-up (ranging from three months to 13.1 years) several clinical examination tests were performed, including standard clinical examinations by the surgeons, CT, and standard hematologic tests. These tests showed new bone formation between bioactive glass granule remnants as well as bone bridging in between the individual granules. Description of remnants in this study implies a slow degradation of S53P4 bioactive glass granules* in vivo*. All patients reported being satisfied with the treatment and good clinical outcomes were observed. Observed complications were not related to the use of S53P4 bioactive glass.

There is no consensus in literature about a gold standard procedure in nasal septal perforation repair [42]. Stoor et al. [23, 24] investigated the possibility of repairing septal perforations with S53P4 bioactive glass discs (220–1300 mm^2^; a thickness of 2 mm). One or two bioactive glass discs were used as graft material in eleven patients and when available crushed autologous cartilage or bone from the operative site was used. The number of patients where cartilage or bone was used was not reported. Treatment with bioactive glass discs was successful in 10/11 [23] and 22/23 patients [24]. In both studies, the perforation in one patient could not be closed due to a near total septum perforation after hypophysis surgery. After a follow-up of 2–37 months, the septal perforations were closed, without observed infections, in both studies. No extrusion of the bioactive glass was observed. Two patients suffered from a small recurrent perforation, repaired during a second operation, without further reperforation [23, 24]. The authors did not report bone ingrowth or degradation of the bioactive glass discs. Although the patient group was small and some details in the reported cases were lacking, the use of S53P4 bioactive glass discs seemed to be a good option for the treatment of septal perforations.

Turunen et al. [25] used bioactive glass granules (0.8–1 mm) in combination with corticocancellous bone chips (average size 1 × 3 × 5 mm), harvested from the patient's iliac crest, for maxillary sinus floor augmentation. As a control, autologous bone chips alone were used for filling the anterior part of the contralateral sinus. Again promising results were obtained as witnessed by histology of biopsies (49–62 weeks postoperatively), scanning electron microscopy (SEM), and energy-dispersive-X-ray analysis. Thicker bone lamellae were found in the bioactive glass in combination with autologous bone group than in the group with only autologous bone chips. Bone quantity did not differ significantly between groups. In the contact areas, bone grew on the glass surface, connecting the granules. The authors concluded that the use of bioactive glass granules combined with autologous bone chips for augmentation of the maxillary sinus floor reduced the amount of bone needed and formed the same quantity of bone in the defect as autologous bone chips alone [25]. This is a very promising finding, but in clinical practice it may be favourable not to use autologous bone at all since it requires a second surgery site with associated complications (e.g., donor site morbidity) and costs [1, 43, 44]. For this reason additional research and clinical evidence on the usage of S53P4 bioactive glass granules in maxillary sinus floor augmentation is needed.

In mastoid obliteration for the treatment of chronic otitis, S53P4 bioactive glass granules were used in three different studies [26–28]. Stoor et al. [28] were the first to describe this treatment, and they did not observe complications due to the bioactive glass granules. The size of the cavity in the mastoid cell area decreased in all seven treated patients. In 2012, both Sarin et al. [26] and Silvola [27] described the use of S53P4 bioactive glass granules in mastoid obliteration. Sarin et al. reported a 92% success rate (success was reported as achieving a dry, smaller, or nonexistent cavity) after a median follow-up period of 34.5 months. Silvola described dry ears in all sixteen patients within a month after obliteration but also absence of symptoms, easy views into the canals, and normal skin in most of the patients. Only two patients with complications were observed. One of the patients needed revision because of ruptured skin. The other patient was revised because of too extensive filling. After revision both patients performed well. Most patients included in this study had a long history of cleaning problems and treatment-resistant otorrhea, but treatment with bioactive glass granules was successful for the reported follow-up period (up to five years postoperatively) [27].

In all aforementioned studies, beside good clinical outcomes, cosmetic results were described to be good as well. In orbital wall reconstruction, eyes were in the correct position postoperatively, and in septal perforation repair the septum was straight. Moreover, all studies described the absence of foreign body responses and infections, which are two major advantages of bioactive glass over other synthetic materials used in head and neck surgery [22]. Absence of infections is thought to be a result of the material's antimicrobial properties [9, 12, 15, 17]. The results of the reported studies indicate that S53P4 bioactive glass is a promising bone substitute material for craniofacial bone graft applications, with high potential in regeneration of lost bone.

## 3. Clinical Application of S53P4 Bioactive Glass in the Treatment of Osteomyelitis

Osteomyelitis is an infection of bone and bone marrow. Currently, osteomyelitis is commonly treated with surgical implantation of polymethyl methacrylate (PMMA) beads, mixed with antibiotics, in the anatomical area of osteomyelitis after extensive debridement and pulse lavage [45]. These antibiotic PMMA beads must be removed by subsequent surgical intervention, usually after two weeks [46, 47]. With the discovery that S53P4 bioactive glass possesses antimicrobial properties [12, 15, 17], the treatment of chronic osteomyelitis by S53P4 bioactive glass granules was clinically investigated (Table 2) [29–32]. Antibacterial properties are a result of a local pH increase that is caused by the exchange of alkali ions with protons in solution (body fluid) [17]. The release of salt ions contributes to a higher osmotic pressure, which is also indicated as an antimicrobial factor [7–9], as illustrated in Figure 1.

Romanò et al. [29, 30] compared the use of bioactive glass granules to two local antibiotic delivery therapies (antibiotic loaded hydroxyapatite with calcium sulphate; a combination of tricalcium phosphate and teicoplanin-loaded demineralised bone matrix) and found comparable results after approximately 22 months. At a mean follow-up of 21.8 months, no recurrent infections were observed in 92.6% of the patients treated with S53P4 bioactive glass granules. Of the patients treated with antibiotic loaded hydroxyapatite and calcium sulphate compounds, 88.9% was infection-free. In 86.3% of the patients treated with a mixture of tricalcium phosphate and an antibiotic loaded demineralized bone matrix, no reinfection was observed. A significant difference was found in wound healing: less prolonged serum wound leakage was observed with the treatment of S53P4 bioactive glass granules [29, 30]. This indicates that the treatment of osteomyelitis with S53P4 bioactive glass granules was at least as successful as the current treatment, if not even better. These findings are important, as it is known that bacteria (also the ones that can cause osteomyelitis) can become resistant to antibiotics [46, 48, 49]. The antimicrobial working mechanism of S53P4 bioactive glass is completely different from the working mechanism of antibiotics, which might make it more reliable in the long run. However, it has yet to be investigated if bacteria can develop resistance against this treatment as well.

Others have also reported successful treatment of chronic osteomyelitis in clinical practice. McAndrew et al. [32] describe a fully treated bone infection in three treated patients. The follow-up at 14 to 21 months showed no radiological evidence of osteomyelitis during this period, with good integration of bioactive glass and surrounding bone. This was also observed in an earlier study by Lindfors et al. [31], who described the successful treatment of chronic osteomyelitis by S53P4 bioactive glass granules in eleven patients. In this study, again, no adverse effects of bioactive glass were observed. The clinical outcome was good or excellent in nine patients (mean follow-up of 24 months). Two cases that did not score good or excellent had complications due to haematoma or because of infections in the muscle flap (no signs of osteomyelitis on X-rays). The reason for the formation of the haematoma could have been attributed to improper filling of the defect with S53P4 bioactive glass granules [31]. Filling the cavity was found to be extremely important, since improper filling could lead to reinfection of the bone as has also been described by Romanò et al. [30]. The other study also had a patient with infection of the muscle flap, with recurrent osteomyelitis after two years as a result. These two cases indicate that not only proper filling but also treatment of the soft tissue surrounding the bone is important. Moreover, another very important issue in the treatment of osteomyelitis is the formation of new blood vessels during the regeneration of the bone, to prevent sepsis [49]. There are indications that S53P4 bioactive glass has angiogenic potential. However, evidence is scarce and is only based on* in vitro* findings [11]. The angiogenic effect could provide a crucial link in the bone healing cascade and remains an important topic for future research.

The success rate of S53P4 bioactive glass granules in the treatment of osteomyelitis is 25/27 [30], 3/3 [32], and 9/11 [31], which provides success in 90% of all reported cases. In contrast, the treatment with gentamicin-PMMA beads of 100 patients suffering from osteomyelitis succeeded in 78% of the patients [47]. It has to be noted that 92% of the patients treated with gentamicin-PMMA beads healed after revisions during the treatment period. Thus the treatment with bioactive glass can be concluded to be at least as effective as the standard procedure but has the additional benefit that it is a single stage procedure, whereas the standard procedure requires two operations. With only one surgical procedure needed there is a smaller chance for occurrence of comorbidities, the hospital stay will be shortened, and healthcare costs will be reduced. Moreover, S53P4 bioactive glass allows remodelling to natural bone over time, which ensures conservation of bone stock. This is important as many of these patients ultimately require additional surgery later in life (e.g., joint replacement). More prospectively gathered clinical data in well-defined study cohorts is needed to determine if S53P4 bioactive glass will replace the antibiotic containing PMMA beads as the current gold standard treatment of osteomyelitis. Most preferably such studies should utilize a randomized controlled trial setup, which will make a direct comparison possible.

## 4. Clinical Application of S53P4 in Spondylodesis and Depressed Tibial Plateau Fractures

Limited clinical results are available for use of S53P4 bioactive glass in instrumented posterior spondylodesis with transpedicular screw fixation. In the treatment of degenerative spondylolisthesis, autogenous bone grafting is still the gold standard procedure [33]. Because of the disadvantages associated with autologous bone harvesting, S53P4 bioactive glass was investigated as a possible alternative treatment (Table 3). Two prospective long-term follow-up studies on S53P4 bioactive glass granules (1-2 mm) and autologous bone grafts as bone graft substitutes for the treatment of unstable lumbar spine burst fractures have been reported [33, 34]. In both studies, autologous bone grafts were used as a control and implanted in the contralateral side. Subjective satisfaction after 11 years of follow-up was better than before treatment in fifteen out of seventeen patients. The results of CT scans indicated good fusion of bone with S53P4, with fusion rates of 71% [34] and 88% [33]. However, these results are poor when compared to those of the autologous bone grafts, which have a fusion rate between roughly 80 and 100% [33, 34]. In the study by Frantzén et al. [33] subjective patient satisfaction was evaluated before and after surgery, resulting in more satisfied patients post-operatively in most cases (only one was unchanged and one worsened). The subjective patient satisfaction in the study by Rantakokko et al. [34] was excellent in two cases, good in five, and fair in three. No poor satisfaction was reported. Visual analogue scale (VAS) pain scores decreased in almost all patients [33, 34]. However, it is hard to correlate these rates solely with the use of bioactive glass since in all patients autologous bone was implanted contralateral to the bioactive glass side.

Since the bioactive glass granules show low fusion rates, it is needed to be concluded that bioactive glass granules, in its current form, cannot be used as a stand-alone solution for posterolateral fusion. Reasons for the bioactive glass not to perform as expected could be the high rotational and compressional forces that are present in the spine. Those forces are not present in other reported applications (e.g., craniofacial applications). High rotational forces in spinal indications have so far not been studied in depth.

Similar to the spine, the tibial plateau is a mostly compressive but still multidirectional load-bearing bone structure. The standard treatment for depressed tibial plateaus is treatment with autologous bone grafts [35]. Only one study was found on the use of S53P4 bioactive glass in the treatment of depressed tibial plateau fractures. Heikillä et al. [35] performed a randomized study to test the applicability of S53P4 bioactive glass granules in tibial fractures. In this study, patients with a depressed unilateral tibial plateau fracture were divided randomly into two groups. One group was treated with S53P4 bioactive glass granules (size 0.83–3.15 mm) and the other with conventional autologous bone grafts. At one-year follow-up, no differences were found between the two groups in clinical examination, functional tests, and radiological examinations. Also in long-term follow-up (up to eleven years), no significant differences between groups were observed based on CT assessment [50]. The results show that S53P4 bioactive glass granules are a possible material to use in tibial plateau fracture treatment. However, the treated patient group was small.

## 5. Clinical Application of S53P4 as Bone Graft Material after Benign Bone Tumour Resection

In the grafting of bone defects that are a result of tumour removal, autologous bone grafts are the standard [51]. Because of aforementioned reasons, bioactive glass has also been studied for treatment of this clinical indication (Table 4) [36–38]. In one randomized trial comparing to autologous bone grafts [36], at twelve months after implantation, small and large cavities could not be observed in CT images anymore in patients grafted with autologous bone, indicating that the bone had completely remodelled. This was significantly different from the bioactive glass group, in which large cavities only started to diminish after twelve months. However, after 24 months no significant difference in the small cavities was observed between the two groups anymore, and after 36 months of follow-up there was no difference in large cavities either. A significant difference was observed in the cortical thickness, which increased more in the bioactive glass group than in the autologous bone graft group. Bone remodelling was slower around bioactive glass, but sclerotic tissue seemed more prone to form in this group, as was observed on plane radiographs. In a revision after two years (due to a residue cyst), it was observed that the granules (the ones that were still present) had incorporated very well with the surrounding bone. Complications were observed in both groups. However, the S53P4 material was not related to these complications.

21 of the 25 patients of a study by Lindfors et al. [36] were followed up in a long-term study (average follow-up of 14 years) [38]. This follow-up confirmed the observations that the newly formed bone with bioactive glass as bone graft was more sclerotic and the cortex was thicker than with the use of autologous bone grafts. Furthermore, no ectopic bone was found in the surrounding soft tissue, which is expected given the fact that bioactive glass is considered osteostimulative and not osteoinductive. Bioactive glass remnants were still visible in six out of eight large bone defects but not in the smaller sized bone defects. This indicates a very slow degradation of the bioactive glass granules when grafted in a large defect. Remodelling of bone was also observed in a case study of a three-year-old child [37]. Already after two years the bone had remodelled to its normal shape and had grown in length. Additionally the range of motion of the proximal and distal interphalangeal joints was 90°, which can be considered as fully restored to the normal range [52].

The reported findings by Lindfors et al. [36–38] showed promising clinical treatment results. Although new bone formation is not as fast as that with the currently used graft (autologous bone), the remodelling of the bone seems better in the long term with denser bone and thicker cortex. In small defects, the differences in bone remodelling between bioactive glass and autologous bone grafts were even smaller. To obtain more confidence in this treatment, a prospective, randomized, multicentre study would be beneficial, since the results that are reported so far are all from the same medical centre with the same surgeons.

## 6. Discussion and Future Recommendations

This review of the clinical evidence for the use of S53P4 bioactive glass showed good results in various clinical indications reporting a follow-up up to fourteen years postoperatively. One of the major advantages of S53P4 bioactive glass compared to autologous bone grafts (the gold standard treatment in most of the discussed applications) is its “off-the-shelf” nature and excellent bone healing capacity. Additionally, it can protect against bacterial adhesion and colonization on its own surface and possesses antimicrobial properties by hampering bacterial growth. In contrast to the two-stage osteomyelitis treatment with antibiotic containing beads, S53P4 bioactive glass offers a one-step treatment solution that eliminates an additional operation procedure which will cost extra time, tools, and added risks for the patients [43]. Moreover, the antimicrobial working mechanism of S53P4 bioactive glass is completely different from that of antibiotics, which might make it more reliable in the long run, with the increasing prevalence of antibiotic resistant bacteria [46, 48, 49].

S53P4 was the only bioactive glass in this review because this composition of bioactive glass is most commonly used in clinical practice to date [9]. However, the development of 45S5 bioactive glass was reported earlier than that of the S53P4 variant [53]. The reason why S53P4 is used more in clinical practice is not clear. The only difference between the two compositions noted in literature is the faster degradation of the 45S5 composition, which could be beneficial in some clinical applications [9]. However, to the knowledge of the authors, the two have never been compared. Moreover, it is not clear how effective the 45S5 composition is in inhibiting bacterial growth. Comparison of the two compositions is needed in order to draw conclusions on their differences and application possibilities.

According to long-term findings, S53P4 bioactive glass degrades slowly with remnants still visible fourteen years after implantation [38]. However, with the use of granules, bone incorporates well and the newly formed bone tissue is stable, also with bioactive glass remnants still present [22, 33, 34, 38]. S53P4 granules are enhancing new bone formation to a larger extend than bioactive glass plates [18]. The main explanation for this might be the intergranular porosity. The S53P4 bioactive glass plates used in orbital floor reconstruction were appropriate for this application since the main function is providing stability. This will also be reached without the full integration into the surrounding bone.

It is noteworthy that in none of the reported papers foreign body reactions or infections were observed in the studied patients, regardless of the clinical application. Some of the studied procedures are susceptible for infections, for example, the treatment of nasal septal perforation. Because of its antimicrobial effects, the treatment of osteomyelitis became a new indication for S53P4 bioactive glass. This is even more beneficial since the current treatment with antibiotics is rapidly becoming problematic due to the rise in antibiotic resistant bacteria. With clinical success rates at least comparable to the gold standard procedure [30, 31, 47], it might become the gold standard treatment for osteomyelitis treatment in the future. More experience with the treatment would be of great benefit, since two studies already have taught us that success is strongly dependent on proper debridement and filling of the osteomyelitic defect [30, 31]. Moreover, it is important to gain more experience since the material choice will always be based on the experience and preference of the surgeons. They will choose a material that they believe will provide the best clinical results and lowest complication rates for the individual patients [39]. Lindfors et al. [37] show that in the treatment of benign bone tumours S53P4 bioactive glass is very promising. Considering all the positive results, S53P4 bioactive glass seems an effective treatment of osteomyelitis and benign bone tumours. For future research, prospective, randomized, multicentre clinical studies will be needed to evaluate and further strengthen the use of bioactive glass in the applications for osteomyelitis treatment and benign bone tumour grafting procedures.

Results obtained in spondylodesis procedures did not match the expectations based on other clinical indications [33, 34]. Current treatment with autologous bone has been proven to be more effective (88% against 100% total fusion rates). Therefore, it is possible that treatment with bioactive glass granules may be less effective in spondylodesis applications. More clinical research is needed to provide further insights into this topic, especially the influence of rotational and compressive forces on the efficacy of S53P4 bioactive glass treatment. However, in a small group of patients treated for a depressed tibial plateau fracture results were good [50], and this would suggest that load-bearing applications are possible as well, as long as the material is well contained and shear forces are low (these conditions are met in the tibia plateau but not in the spine). Thus, further research is needed to study the load-bearing capacity of bioactive glass and to delineate conditions where it may or may not be used safely.

To date, evidence on the* in vivo* angiogenic potential of S53P4 bioactive glass has not been published, to the knowledge of the authors. As indicated in literature, angiogenesis is a very important process in bone repair [54]. Especially in large bone defects, lack or slow (neo)vascularization may result in necrosis at the central region of the bone grafts, leading to its ultimate failure [54]. The positive long-term results of the clinical trials with S53P4 bioactive glass in large defects suggest that vessels had grown in, to provide nutrients and oxygen to the cells within the material in order to form new bone. However, no direct evidence is reported. Neovascularization could provide a crucial link in the bone healing cascade and remains an important topic for future research.

Another factor that should be investigated is the influence of granule size in the various applications. In a rabbit model, it has been reported that bone growth was significantly more abundant in bone defects filled with granules of 0.68–0.8 mm than with 0.2–0.25 mm sized granules [55]. However, these granule sizes were not used in the reported clinical applications that were discussed in this review. No differences due to granule size have been reported in human bone repair studies so far.

## 7. Conclusions

This literature review provides evidence that S53P4 bioactive glass can be an effective treatment of bone defects in various clinical situations. Long-term follow-up studies reported excellent results. Although resorption is usually not completely accomplished, the application of S53P4 bioactive glass in craniofacial surgery applications and grafting of benign bone tumour defects could be beneficial, especially when autologous bone grafting is risky or impossible.

Treatment of osteomyelitis with S53P4 bioactive glass is safe and effective even in one-stage treatment options, without the addition of local antibiotics. Adequate debridement, proper defect filling, and adequate containment of the bioactive glass granules are essential. More clinical and health economic cost-effectiveness data is needed to determine if S53P4 bioactive glass can replace antibiotic containing PMMA beads as the current standard of osteomyelitis, most preferably utilising randomized controlled trials.

## Figures and Tables

**Table 1:** Summary of reviewed publications with S53P4 bioactive glass used in craniofacial surgery procedures.

Reference	Clinical indication	Number of treated patients with S53P4 implants	Application form	Number of successful treatments	Complications related to S53P4 implant	Study design	Follow-up period [months]	Examinations during follow-up
Suominen and Kinnunen [18]	Facial reconstructions	36 sites in 13 patients	Granules (0.63–0.8 and 0.8–1 mm) and plates (8 × 10–15 × 29 mm, 1.5, 2.0, 2.5, or 3.0 mm thick)	36	1 reoperation for repositioning of orbital roof	Prospective single centre cohort study	12 (average, range: 6–26)	Clinical examination, radiographs, and QCT

Aitasalo et al. [19]	Orbital floor reconstructions of blowout fractures and zygomaticomaxillary fractures	34	Plates in 3 different sizes (diameter: 20, 25, or 30 mm, 1–1.5 mm thick)	33	1 removal due to incorrect size	Retrospective single centre cohort study	10.9 (average, range: 6–12)	Clinical examination by an ear, nose, and throat surgeon, an ophthalmologist, and a radiologist. Laboratory tests for infection, liver and kidney functions

Peltola et al. [20]	Orbital floor reconstructions of blowout fractures, zygomaticomaxillary fractures, and tumour removal	43	Plates (sizes not reported)	40	3 reoperations due to inappropriate size and shape	Retrospective single centre cohort study	24	Clinical examination by the surgeon, ophthalmologist, examination of CT and MRI images, and laboratory tests for infection and kidney function

Stoor et al. [21]	Orbital floor reconstructions of blowout factures	20	Drop shaped in 2 sizes (1.5 mm thick and 31 × 25 mm or 34 × 26 mm)	20	None	Prospective single centre cohort study	32 (average, range: 6–71)	Clinical examination by the surgeon, examination CT and MRI

Peltola et al. [22]	Frontal sinus obliteration	42	Granules (0.5–0.8 and 0.8–1.0 mm)	39	None, but 2 reobliteration cases due to mucocele 1 reobliteration due to insufficient closure of the nasofrontal duct	Prospective single centre cohort study	73.2 (average, range: 3–13.1)	Clinical evaluation by the surgeons, examination by CT

Stoor et al. [23]	Septal perforation repair	11	Disks (200–1300 mm^2^, 2 mm thick)	8	1 near total septum perforation could not be closed 2 small recurrent perforations	Prospective single centre cohort study	Range: 2–37	Clinical examination not reported

Stoor and Grénman [24]	Septal perforation repair	23	Disks (200–1300 mm^2^, 2 mm thick)	22	1 near total septum perforation could not be closed 5 reoperations because of a small recurrent perforation: closed with bioactive glass, successfully	Prospective single centre cohort study	28 (average, range: 12–68)	Clinical examinations

Turunen et al. [25]	Maxillary sinus floor augmentation	17	Granules (0.8–1.0 mm) mixed with autologous bone chips	17	None	Prospective single centre cohort study	17 (average, range: 7–30)	Examination of biopsies by SEM, EDXA and histologically

Sarin et al. [26]	Mastoid obliteration	26	Plates and granules (sizes not reported)	21	1 reoperation due to inadequate fascia coverage 2 postoperative otorrhea cases which were debrided 2 ears which were not dry	Prospective single centre cohort study	42.5 (average, range: 1–182)	Clinical examinations

Silvola [27]	Mastoid obliteration	16	Granules (0.5–0.8 and 0.8–1.0 mm)	14	1 revision due to ruptured skin 1 meatoplasty because of too extensive filling	Prospective single centre pilot study	26 (average, range: 7–48)	Clinical outcome obtained by a grading system

Stoor et al. [28]	Mastoid obliteration	7	Granules (0.5–0.8 mm)	6	1 infection (related to conservative treatment instead of the S35P4)	Prospective single centre case study	57 (average, range: 22–98)	Clinical examinations, CT imaging (1 patient) Laboratory tests for infection and kidney functions

**Table 2:** Summary of reviewed publications with S53P4 bioactive glass as treatment for chronic osteomyelitis.

Reference	Clinical indication	Number of treated patients with S53P4 implants	Application form	Number of successful treatments	Complications related to S53P4 implant	Study design	Follow-up period [months]	Examinations during follow-up
Romanò et al. [29, 30]	Chronic osteomyelitis	27	Granules (size not reported)	25	2 recurrences of infection (with 1 fracture)	Retrospective single centre cohort study	21.8 (average, range: 12–36)	Clinical and laboratory evaluation and radiographs

Lindfors et al. [31]	Chronic osteomyelitis	11	Granules (0.5–0.8, 0.8–1.0, 1.0–2.0, and 2.0–3.15 mm)	10	1 recurrence due to improper filling of the cavity	Retrospective multicentre cohort study	24 (average, range: 10–38)	Clinical examinations and radiological evaluation

McAndrew et al. [32]	Chronic osteomyelitis	3	Granules (size not reported)	3	None	Retrospective single centre case study	17.3 (average, range: 14–21)	Radiological, haematological, and biochemical examinations

**Table 3:** Summary of reviewed publications with S53P4 bioactive glass for spondylodesis or treatment of depressed tibial plateau fractures.

Reference	Clinical indication	Number of treated patients with S53P4 implants	Application form	Number of successful treatments	Complications related to S53P4 implant	Study design	Follow-up period [months]	Examinations during follow-up
Frantzén et al. [33]	Spondylodesis	17	Granules (1.0–2.0 mm)	15	1 subjective outcome was unchanged after 132 months 1 subjective outcome was worse after 132 months VAS score at 132 months: 3.5 (range: 0–8)	Prospective single centre cohort study	132	Clinical examination, VAS pain score list, subjective satisfaction grades, and examinations on CT, MRI, and DEXA

Rantakokko et al. [34]	Spondylodesis	10	Granules (size not reported)	10	3 subjective outcomes were fair after 120 months 5 were good after 120 months 2 were excellent after 120 months VAS score at 120 months: 1.0 (range: 1–4)	Prospective single centre cohort study	120	Clinical examination, subjective patient satisfaction, VAS pain score and Oswestry disability questionnaires, and CT and DEXA evaluations

Heikkilä et al. [35]	Depressed lateral tibial plateau fractures	14	Granules (0.83–3.15 mm)	14	None	Prospective single centre randomized study	12	Clinical examination by orthopaedic surgeons, CT evaluation, and subjective and functional evaluations

**Table 4:** Summary of reviewed publications with S53P4 bioactive glass as bone graft material in benign bone tumour treatment.

Reference	Clinical indication	Number of treated patients with S53P4 implants	Application form	Number of successful treatments	Complications related to S53P4 implant	Study design	Follow-up period [months]	Examinations during follow-up
Lindfors et al. [36]	Grafting of benign bone tumours	14	Granules (1.0–2.0 and 3.15–4.0 mm)	11	1 reoperation due to a residual cyst 2 fractures due to not following immobilization advice	Prospective single centre randomized study	36	CT and X-ray evaluation and blood sample examinations

Lindfors [37]	Recurrent aneurysmal bone cyst	1	Granules (0.5–0.8 mm)	1	None	A single case study	24	X-ray examination and clinical evaluation on function and growing

Lindfors et al. [38]	Grafting of benign bone tumours	11	Granules (1.0–2.0 and 3.15–4.0 mm)	11	None	Prospective single centre randomized study	168 (average, range: not reported)	Clinical examination, X-ray, MRI, and CT evaluation
